# A Descriptive Study of Atherogenic Indices in Patients Admitted to a Tertiary Care Hospital

**DOI:** 10.7759/cureus.32231

**Published:** 2022-12-05

**Authors:** Saranya Dharmaraj, Sujatha Rajaragupathy, Saju Denishya

**Affiliations:** 1 Biochemistry, Employees' State Insurance Corporation (ESIC) Medical College, Hyderabad, IND; 2 Biochemistry, PSG Institute of Medical Sciences and Research, Coimbatore, IND

**Keywords:** atherogenic indices, hospitalization, high density lipoproteins, diabetes mellitus, cholesterol, cardiovascular diseases

## Abstract

Introduction

Atherogenic indices, as common factors implicated in the pathogeneses of atherogenic dyslipidemias and cardiometabolic disorders, provide inexpensive and less invasive aids for assessing the prognosis of hospitalized patients. Hence, we evaluate the atherogenic index profiles of patients admitted to a tertiary care hospital and correlate them with comorbidities, statin use, and duration of hospital stay.

Methodology

This cross-sectional study included 412 hospitalized patients aged >18 years undergoing lipid profiling, irrespective of their diagnosis. Their atherogenic indices were calculated from their lipid profile parameters and correlated with their comorbidities, statin use, and duration of hospital stay. Statistical analysis was done using the Mann-Whitney U test and Spearman’s rank correlation coefficient tests, with a p-value of <0.05 indicating statistical significance.

Results

The participating cohort showed a mean age of 56.01±13.32 years. Nearly 63.0% of these had diabetes mellitus, 52.0% had hypertension, 34.0% had coronary artery disease, 16.0% had a cardiovascular accident, and 16.5% reported statin use. There was no significant difference in the distribution of any of the atherogenic indices over any of the comorbidities like diabetes mellitus, hypertension, coronary artery disease, or statin use, except for the non-high-density lipoprotein cholesterol distribution, which was significantly associated with coronary artery disease (p-value = 0.0112) and statin use (p-value = 0.0057). Atherogenic indices were not correlated with the duration of hospital stay (p-value > 0.05).

Discussion

This study suggests that non-high-density lipoprotein cholesterol may serve as an indicator of coronary artery disease and statin use. However, other atherogenic indices may not serve as reliable predictors of the duration of a hospital stay.

## Introduction

Cardiovascular disease is the leading cause of mortality, accounting for 17.7 million deaths, with India contributing to one-fifth of these fatalities [1,2]. Over the years, atherogenic dyslipidemia has been identified as being strongly associated with an increased risk of cardiovascular disease (CVD) and metabolic syndromes, mainly related to lifestyle and pro-inflammatory factors. Diabetes mellitus (DM), hypertension (HTN), coronary artery disease (CAD), and cerebrovascular accidents (CVA) are all exacerbated by the pro-oxidative and pro-thrombotic state associated with insulin resistance [3, 4]. 

This warrants exploring and improving the predictive capacity of lipid profiles using atherogenic indices (AI) in prognosticating cardiometabolic comorbidities common in hospitalized patients. AI includes the thermogenic index of plasma (AIP), Castelli risk index I and II (CRI-I, CRI-II), atherogenic coefficient (AC), and non-HDL cholesterol (NHC), which are known independent risk factors for cardiovascular risk [5-7]. Studies have shown that AI calculated from the parameters of a lipid profile has been found to have better cardiovascular predictive capacity than individual lipid parameters alone [8].

The development of reliable AI parameters to predict risks and disease progression in hospitalized patients with or without comorbidities would provide affordable and minimally invasive assistance in reducing the public health burden. Once conclusively identified, these parameters can serve as markers of hospital stay duration and hospitalization outcomes. However, these postulated relationships need to be verified before relying on them completely. This is the first research of its kind, to the best of the authors' knowledge, that covers a wide range of AI and examines their relationship with relevant factors. Hence, this study was designed with the aim to evaluate the AI profiles of patients admitted to a tertiary care hospital and correlate them with co-morbidities, statin use, and duration of hospital stay (DOHS).

## Materials and methods

This cross-sectional study was conducted at the PSG Institute of Medical Sciences and Research, a tertiary care hospital in Coimbatore, Tamil Nadu, India, for six months after obtaining ethical clearance from the Institutional Human Ethics Committee (PSG/IHEC/2021/Appr/Exp/160), dated July 2, 2021. The study followed convenient sampling, including 412 hospitalized patients aged >18 years who underwent lipid profiling irrespective of their diagnosis. The study excluded critically ill patients, since lipid values are spurious in these patients during the acute phase of illness, and those in whom lipid profiling was not done. Informed consent was obtained from each patient included in the study.

For all patients, demographic data (age, gender), medical history, DOHS, and laboratory data regarding lipid profile were obtained from the Laboratory Information System (LIS), consisting of total cholesterol (TC), triglyceride (TG), high-density lipoprotein cholesterol (HDL-C), and low-density lipoprotein cholesterol (LDL-C) levels. The parameters were analyzed in a fasting blood sample collected in serum tubes and estimated using dedicated kits and reagents.

The following atherogenic indices were then calculated and analyzed:

Atherogenic index of plasma (AIP) = log(TG/HDL-C); Castelli risk index I (CRI-I) = TC/HDL-C; Castelli risk index II (CRI-II) = LDL-C/HDL-C; Atherogenic coefficient (AC) = (TC - HDL-C)/HDL-C; Non-HDLC (NHC) = TC - HDL-C

The data were analyzed using R software version 4.1.2 and Microsoft Excel. Categorical variables are given in the form of a frequency table. Continuous variables are given in mean ± standard deviation (SD) or median (minimum, maximum) form. Mann-Whitney A U test is used to compare distributions of AI with the presence of co-morbidities (DM, HTN, CAD, and CVA) and statin use. Spearman’s rank correlation coefficient is used to check the correlation of AI with the DOHS. A p-value ≤0.05 indicates statistical significance.

## Results

This study analyzed the AI of 412 participants with a mean age of 56.01 ± 13.32 years and a male-to-female ratio of 1.7:1. Table 1 and Table 2 present the descriptive statistics for the participants. A history of DM was reported in 63.8% of the participants, HTN in 52.2%, CAD in 34.0%, CVA in 16.0%, and statin use in 16.5% of the participants.

**Table 1:** Descriptive statistics of categorical variables

Variables		Number of subjects (%) [n=412]
Age (years)	≤20	1 (0.24%)
	21-40	56 (13.59%)
	41-60	204 (49.51%)
	61-80	142 (34.47%)
	81-100	9 (2.18%)
Gender	Female	151 (36.65%)
	Male	261 (63.35%)
Diabetes mellitus	No	149 (36.17%)
	Yes	263 (63.83%)
Hypertension	No	195 (47.33%)
	Yes	215 (52.18%)
Coronary artery disease	No	272 (66.02%)
	Yes	140 (33.98%)
Cerebrovascular accident	No	344 (83.5%)
	Yes	66 (16.02%)
Statin use	No	340 (82.52%)
	Yes	68 (16.5%)

**Table 2:** Descriptive statistics of continuous variables SD: standard deviation; IQR: interquartile range

Variables	N	Mean ± SD	Median (IQR range)
Age (years)	412	56.01 ± 13.32	56 (47, 65)
Duration of hospital stay (DOHS) (days)	412	6.59 ± 5.07	5 (3, 8)
Total cholesterol (TC) (mg/dL) (Ref: <200)	411	150.76 ± 48.22	149 (117.5, 181)
Triglyceride (TG) (mg/dL) (Ref: <150)	409	138.5 ± 82.61	119 (87, 164)
High-density lipoprotein cholesterol (HDL-c) (mg/dL) (Ref: 40-60)	409	38.63 ± 23.89	34 (27, 43)
Low-density lipoprotein cholesterol (LDL-c) (mg/dL) (Ref: <100)	409	92.6 ± 44.94	90 (58, 125)
Atherogenic index of plasma (AIP)	409	0.56 ± 0.32	0.56 (0.34, 0.73)
Castelli risk index I (CRI-I)	409	4.78 ± 2.57	4.24 (3.39, 5.6)
Castelli risk index II(CRI-II)	409	2.85 ± 1.53	2.7 (1.85, 3.68)
Atherogenic coefficient (AC)	409	3.78 ± 2.57	3.24 (2.39, 4.6)
Non-high-density lipoprotein cholesterol (NHC)(mg/dL)	409	112.11 ± 50.55	109 (81, 144)
Very low-density lipoprotein (VLDL) (mg/dL)	409	27.7 ± 16.52	23.8 (17.4, 32.8)
Random blood glucose (RBS) (mg/dL)	268	191.74 ± 105.97	157 (121.5, 229.25)
Glycated hemoglobin (HbA1c) (%)	309	8.01 ± 2.55	7.1 (6, 9.6)
Thyroid-stimulating hormone (TSH) (micro IU/ml)	314	2.83 ± 5.92	1.91 (1.07, 3.35)

The NHC distribution showed a significant association with CAD (p-value = 0.0112) and statin use (p-value = 0.0057), and no significant difference in the distribution of AIP, CRI-I, CRI-II, and AC over any of the comorbidities or statin use was noted (Table 3).

**Table 3:** Comparison of atherogenic indices with co-morbidities and statin use *indicates statistical significance; AC = atherogenic coefficient; AIP = atherogenic index of plasma; CAD = coronary artery disease; CRI = Castelli risk index; CVA = cardiovascular accident; DM = diabetes mellitus; HTN = hypertension; MW = Mann-Whitney U test; NHC = non-high-density lipoprotein cholesterol.

Variables		N	AIP median (IQR range)	CRI-I median (IQR range)	CRI-II median (IQR range)	AC median (IQR range)	NHC median (IQR range)
DM	No	149	0.55 (0.29, 0.7)	4.49 (3.48, 5.44)	2.96 (2.02, 3.77)	3.49 (2.48, 4.44)	120 (84, 144)
	Yes	263	0.57 (0.38, 0.73)	4.14 (3.28, 5.62)	2.51 (1.74, 3.6)	3.14 (2.28, 4.62)	105 (79, 143)
p-value			0.3073	0.2653	0.6002	0.3919	0.9226
HTN	No	195	0.57 (0.38, 0.73)	4.41 (3.48, 5.94)	2.72 (2.03, 3.94)	3.41 (2.48, 4.94)	111 (84, 147)
	Yes	215	0.53 (0.31, 0.72)	4.13 (3.13, 5.41)	2.64 (1.64, 3.59)	3.13 (2.13, 4.41)	107 (81, 140)
p-value			0.1853	0.1229	0.7829	0.3384	0.2302
CAD	No	272	0.55 (0.31, 0.73)	4.24 (3.39, 5.62)	2.6 (1.73, 3.6)	3.24 (2.39, 4.62)	109 (81, 143)
	Yes	140	0.56 (0.38, 0.73)	4.3 (3.37, 5.56)	2.9 (2, 3.85)	3.3 (2.37, 4.56)	109.5 (80.5, 145.5)
p-value			0.1037	0.0805	0.1466	0.6234	0.0112*
CVA	No	344	0.55 (0.33, 0.72)	4.18 (3.3, 5.47)	2.7 (1.74, 3.75)	3.18 (2.3, 4.47)	108 (81, 143)
	Yes	66	0.59 (0.36, 0.78)	4.42 (3.56, 5.8)	2.59 (2, 3.59)	3.42 (2.56, 4.8)	120 (90, 155)
p-value			0.1853	0.1229	0.7829	0.3384	0.2302
Statin	No	340	0.56 (0.32, 0.72)	4.14 (3.3, 5.47)	2.6 (1.73, 3.59)	3.14 (2.3, 4.47)	108 (79, 141)
	Yes	68	0.54 (0.36, 0.72)	4.49 (3.51, 5.98)	3.1 (2.23, 4.07)	3.49 (2.51, 4.98)	128.5 (94.5, 155)
p-value			0.1805	0.2241	0.7227	0.1788	0.0057*

Spearman’s rank correlation test found no significant correlation between AI and the duration of hospital stay (p-value>0.05) (Table 4).

**Table 4:** Correlation of atherogenic indices with the duration of hospital stay SP = Spearman’s rank correlation test.

Atherogenic index	Correlation coefficient	p-value
Atherogenic index of plasma (AIP)	0.0456	0.3573
Castelli risk index I (CRI-I)	0.0456	0.3573
Castelli risk index II (CRI-II)	-0.0236	0.634
Atherogenic coefficient (AC)	-0.0843	0.0886
Non-high-density lipoprotein cholesterol (NHC)	-0.0459	0.3547

## Discussion

Considering the common factors involved in the pathogenesis of atherogenic dyslipidemias and cardiometabolic diseases, atherogenic indices (AI) could provide affordable and minimally invasive aids for determining the prognosis of hospitalized patients. Hence, the present study was designed to evaluate the AI profiles of patients admitted to a tertiary care hospital and correlate them with comorbidities, statin use, and DOHS.

This study finds literary support in a previous study where no significant correlation was seen between DOHS and AI or lipid profiles among hospitalized cardiovascular patients, but NHC, along with CRI-I and AC, was significantly related to the risk of stroke [5]. Whereas in the present study, NHC was found to be associated with CAD and statin use, but not stroke (CVA). Furthermore, CRI-I and AC were not found to be related to any cardiovascular or diabetic condition in the present study.

Moreover, contrary to the present research, Cai et al. found AIP to be an independent and strong predictor of CAD (odds ratio 1.782, 95% confidence interval 10.490-2.131, p-value<0.001) [9]. Niroumand et al., too, determined AIP to be useful for routine monitoring of CVD, especially in people with other cardiovascular risk factors [7]. According to Wang et al., the diagnostic cutoff AIP value for predicting CAD was 2.035, with a specificity of 61.8% and a sensitivity of 76.4% (95 % CI: 0.717-0.750, p-value<0.01), suggesting AIP is a viable biomarker for CAD prevention [10]. The current study, on the other hand, revealed no significant relationship between AIP and any cardiometabolic disorder (p-value > 0.05).

Conflicting with the findings of the present research, Bhardwaj et al. found all three AI (AIP, CRI, and AC) to be significantly greater in angiographically confirmed CAD patients compared to age- and sex-matched healthy volunteers (p-value<0.001) [11]. They went on to suggest that these indices can contribute significantly to CAD-risk estimation, especially in the absence of marked derangement of absolute lipid parameters or sufficient resources, but these findings could not be confirmed in the current study [11]. The reason for the discrepancy could be due to the inclusivity of all hospitalized patients and the low representation of CAD patients.

Although AIP theoretically reflects the balance between atherogenic risk and protective factors and has been found suitable and statistically reliable for quantifying the response to therapeutic intervention in diabetic patients, this relationship was not reflected in the present research [12]. A multivariate logistic model analysis by Frohlich et al also reported AIP to be a significant predictor of angiographically defined CAD, in contrast to the present study [13]. Moreover, Wu et al noted NHC to be a significant predictor of ischemic stroke risk (HR: 1·53; 95% CI: 1·24-1·88), unlike the present research, whereby NHC was seen to be associated with CAD and statin use but not with stroke [14].

As a result, the current findings are largely at odds with the extensive literature that supports AI as a valid indicator of cardiometabolic risk and DOHS. Despite the fact that the current study was able to assess the atherogenic profile of hospitalized patients regardless of diagnosis, it was unable to find a link between AI and comorbidities, statin use, or DOHS. The only AI parameter that may accurately predict CAD or statin usage is NHC [15].

Increased levels of low-density lipoprotein cholesterol (LDL-C) have consistently been linked to an increased risk of cardiovascular disease development and mortality [15]. The potential value of non-HDL-C levels as a predictor of cardiovascular mortality has been proven in the literature [16]. The simplicity with which non-HDL-C levels may be measured compared to LDL-C levels is a practical basis for recommending it as a risk assessment tool.

However, this study has certain shortcomings, such as a small sample size restricted to one healthcare facility and a limited range of medical conditions and drugs. The inclusion of all hospitalized patients is a major drawback; however, the study aims to describe the indices in all hospitalized patients. Hence, significant conclusions could not be drawn based on the results of the study.

## Conclusions

NHC is a reliable predictor of CVD mortality, and an elevated NHC level is associated with an increased risk for the development of CVD. From the results of the present study, it can be concluded that AI may not serve as a reliable predictor of DOHS and is not associated with co-morbidities or statin use in hospitalized patients, except for NHC. More multicentric research addressing a wider variety of comorbidities, drugs, and outcomes is necessary to derive definitive conclusions and apply the practical uses of AI in identifying and preventing cardiometabolic disorders.
